# Does Nebulin Make Tropomyosin Less Dynamic in Mature Myofibrils in Cross-Striated Myocytes?

**DOI:** 10.4172/2157-7099/1000239

**Published:** 2014-04-25

**Authors:** DK Dube, J Wang, Y Fan, JM Sanger, JW Sanger

**Affiliations:** Department of Cell and Developmental Biology, SUNY Upstate Medical University, 750 East Adams Street, Syracuse, NY, USA

**Keywords:** Nebulin, Tropomysin, Myofibrils, Premyofibril, Myofibrillogenesis, Cardiomyocytes

## Abstract

Myofibrils in vertebrate cardiac and skeletal muscles are characterized by groups of proteins arranged in contractile units or sarcomeres, which consist of four major components – thin filaments, thick filaments, titin and Z-bands. The thin actin/tropomyosin-containing filaments are embedded in the Z-bands and interdigitate with the myosin-containing thick filaments aligned in A-bands. Titin is attached to the Z-band and extends upto the middle of the A-Band. In this mini review, we have addressed the mechanism of myofibril assembly as well as the dynamics and maintenance of the myofibrils in cardiac and skeletal muscle cells. Evidence from our research as well as from other laboratories favors the premyofibril model of myofibrillogenesis. This three-step model (premyofibril to nascent myofibril to mature myofibril) not only provides a reasonable mechanism for sequential interaction of various proteins during assembly of myofibrils, but also suggests why the dynamics of a thin filament protein like tropomyosin is higher in cardiac muscle than in skeletal muscles. The dynamics of tropomyosin not only varies in different muscle types (cardiac vs. skeletal), but also varies during myofibrillogenesis, for example, premyofibril versus mature myofibrils in skeletal muscle. One of the major differences in protein composition between cardiac and skeletal muscle is nebulin localized along the thin filaments (two nebulins/thin filament) of mature myofibrils in skeletal muscle cells, but which is expressed in a minimal quantity (one nebulin/50 actin filaments) in ventricular cardiomyocytes. Interestingly, nebulin is not associated with premyofibrils in skeletal muscle. Our FRAP(Fluorescence Recovery After Photobleaching) results suggest that tropomyosin is more dynamic in premyofibrils than in mature myofibrils in skeletal muscle, and also, the dynamics of tropomyosin in mature myofibrils is significantly higher in cardiac muscle compared to skeletal muscle. Our working hypothesis is that the association of nebulin in mature myofibrils renders tropomyosin less dynamic in skeletal muscle.

## Introduction

**Figure 1:**
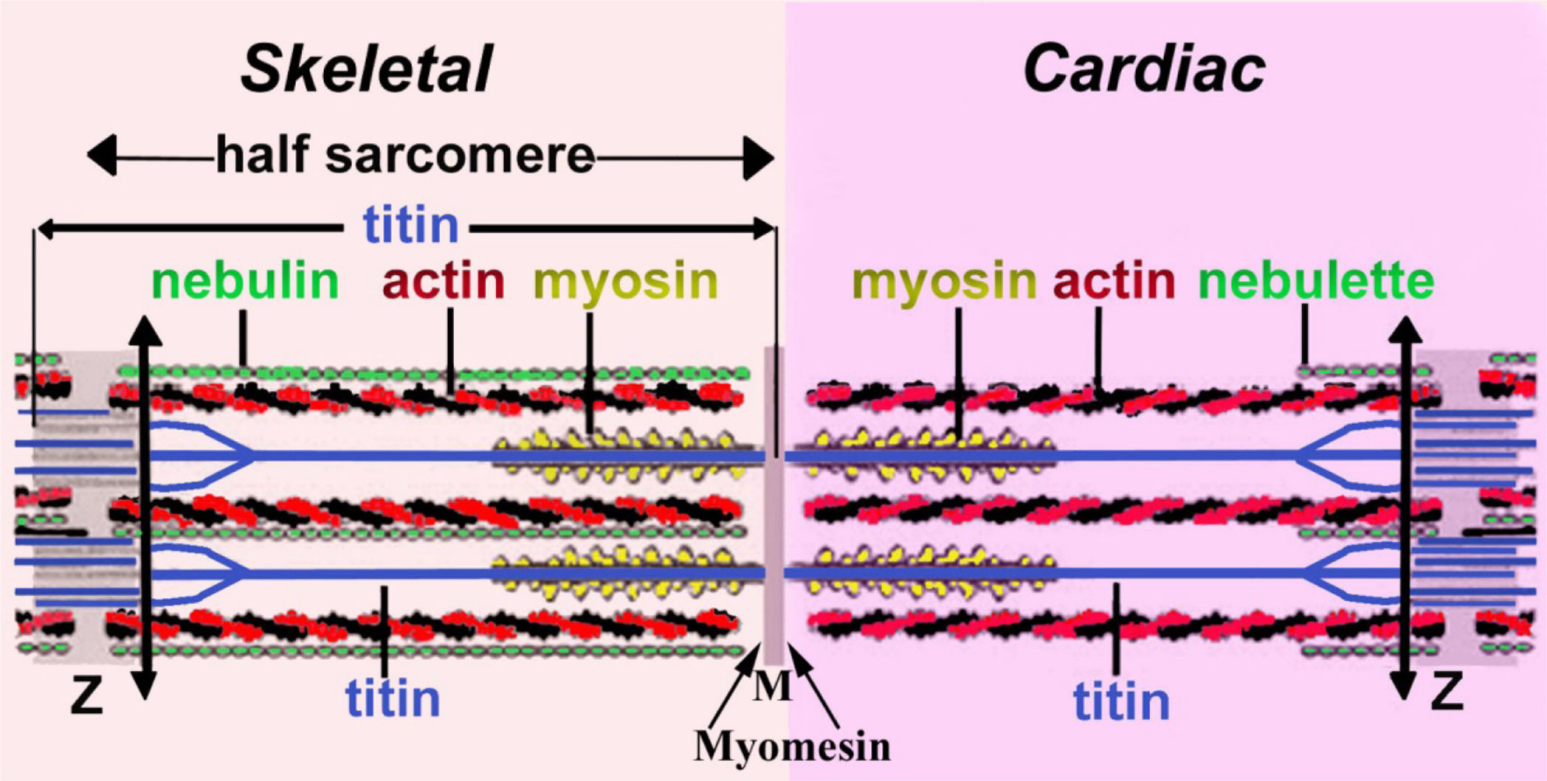
Diagram comparing half sarcomeres of skeletal versus cardiac muscles in vertebrates. Note the major difference between the individual long length of nebulin molecules that run along the entire length of the thin filament in skeletal muscles versus the much shorter lengths of nebulette that extend for a short length of the thin filaments in cardiac muscles. Modified from a previous published Figure in the Journal of Cell Biology by Sanger and Sanger [2001] [30].

There are three types of vertebrate muscles – skeletal muscle, cardiac muscles, and smooth muscle. Skeletal muscle contraction is essential for the movement and cardiac and smooth muscle contraction are required for blood flow throughout the body. Myofibrils are the contractile units of the two cross-striated cardiac and skeletal muscle cell types and they are composed of sarcomeres, which contain actin filament (thin filament) and filament containing myosin (thick filament). Thick and thin filaments slide past one another to generate the forces of muscle contraction. Myosin-based thick filaments are uniform in length, have myosin heads in bipolar orientations and registered in the middle of sarcomeres. Actin-based thin filaments are oriented in opposite directions at each end of a sarcomeric unit (Figure 1). Such an arrangement is essential for the production of contractile forces by unidirectional movement of the myosin motors. Z-bands separate each sarcomere unit and anchor the barbed ends (or rapidly growing end) of thin filaments via α-actinin crosslinking. To the contrary, the pointed ends (or slow growing ends) of actin filaments are free to slide between the surrounding thick filaments. The length of thin filaments in vertebrate skeletal muscle is uniform where as it is variable in cardiac muscles. The mechanism(s) by which the uniform length of the thin and thick filaments is (are) maintained is yet to be resolved.

## Mechanism of Myofibrillogenesis

Despite the differences in functional specialization that cardiac and skeletal muscles acquire during development, the basic process of assembly of proteins into myofibrils appears to follow similar steps [1–3]. Several models have been proposed to provide a framework for understanding the increasing data on new myofibrillar proteins and their localizations during muscle development. Currently, four models that seek to explain how the assembly occurs in vertebrate cross-striated muscles have been proposed [2]. The four models hypothesize: (1) stress fiber-like structures as templates for the assembly of myofibrils; (2) assembly in which the actin filaments and Z-bands form subunits independently from A-band subunits, with the two subsequently stitched together by titin filaments to form a myofibril; (3) premyofibrils and nascent myofibrils as precursors of mature myofibrils; and (4) assembly occurring without any intermediary structures. The premyofibril model, proposed by Sanger et al. [1–3], is considered here as it would explain better the differences in dynamics of myofibrillar proteins in cardiac vs. skeletal muscle cells under a variety of different conditions.

### Premyofibril model

**Figure 2:**
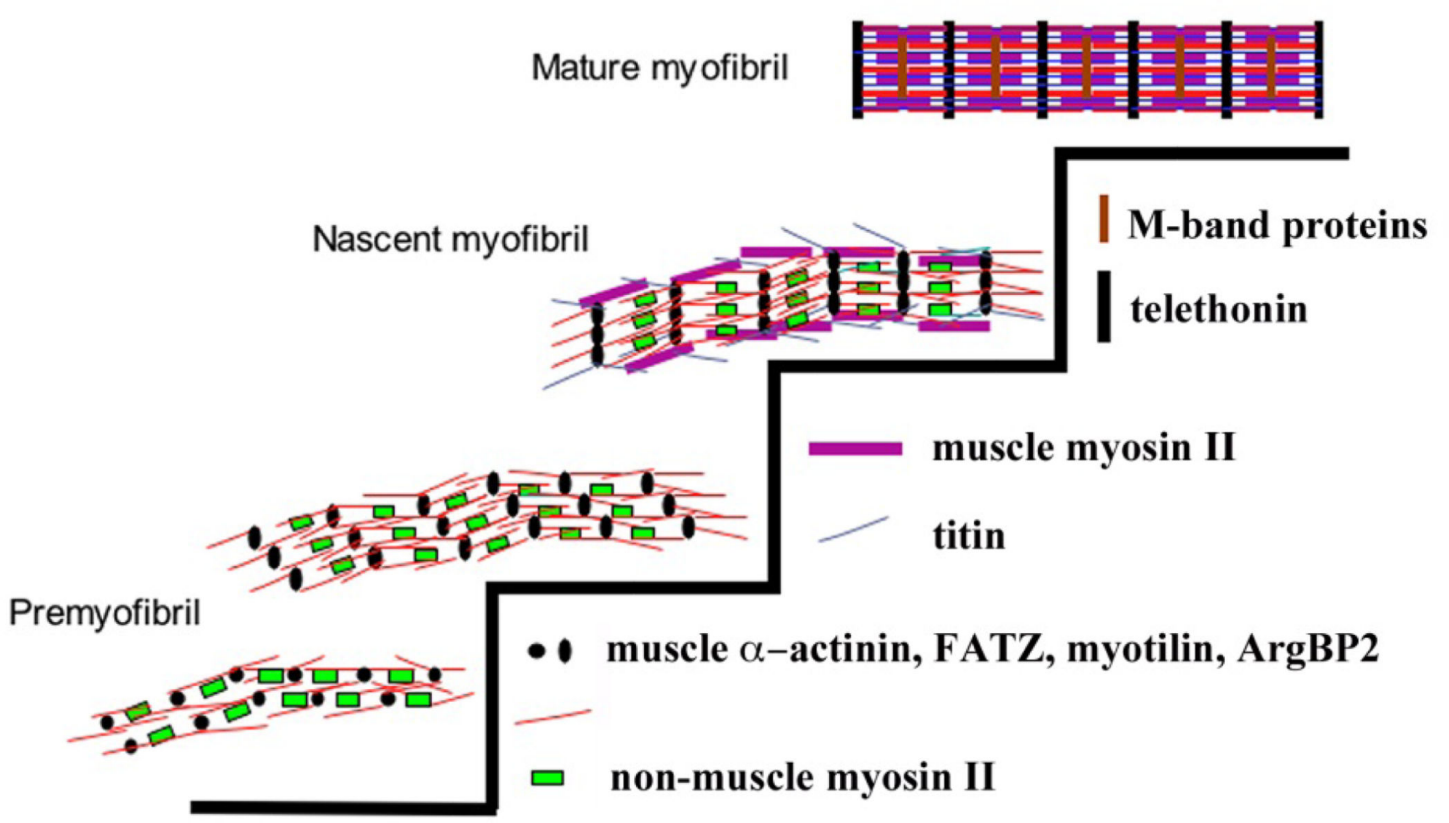
Diagram of the premyofibril model for de novo myofibrillogenesis: premyofibrils to nascent myofibrils to mature myofibrils. Modified from a previous published figure in the Journal of Cell Motility and Muscle Research by Sanger et al (2005) [35.]

Rhee et al. [4] proposed a 3-step model of myofibrillogenesis based on immunolocalization studies in cultured chicken cardiomyocytes fixed and stained after different times in culture (Figure 2). In this model of myofibrillogenesis, the premyofibril forms at or near the spreading plasma membrane surface. It is characterized by α-actinin containing Z-bodies, short filaments of non-muscle myosin IIB and actin filaments attached to the Z-bodies. Z-bodies initially appear as discrete sarcomeric α–actinin aggregates along the premyofibrils. A transition stage exists between pre-myofibrils and mature myofibrils termed nascent myofibrils. The addition of titin, gradual growth and fusion of the Z-bodies into Z-bands, and the incorporation of overlapping muscle myosin filaments mark this stage. The nascent myofibrils thus contain both muscle myosin II and non-muscle myosin IIB, whereas mature myofibrils contain only muscle myosin II but not non-muscle myosin IIB. The mature myofibril is marked by the loss of nonmuscle myosin II, the appearance of thick filament binding proteins, i.e., myomesin and Myosin Binding Protein–C, and the alignment of myosin filaments to form A-bands. Recently, Liu et al. [5] elucidated the mechanism of thick filament assembly onto premyofibrils in living neonatal cardiomyocytes. In this study, the authors concluded that myosin filaments are assembled onto premyofibrils laterally to form nascent myofibrils, which supports our three stage premyofibril model for the formation of mature myofibrils [1,2].

Premyofibril assembly starts at the spreading edges of muscle cells. The premyofibrils are composed of minisarcomeres that contain sarcomeric proteins in the alpha-actinin enriched Z-bodies, and attached thin filaments (F-actin and their associated proteins tropomyosin, and troponins. Nonmuscle myosin II filaments are present in the mini A-bands of the premyofibrils. Z-bodies in adjacent fibrils align in nascent myofibrils, forming beaded Z-bands that transform into Z-bands in mature myofibrils. Titin molecules and muscle myosin II thick filaments are first detected in nascent myofibrils. The thick filaments in the nascent myofibrils are not aligned, but are in an overlapped pattern, exhibiting solid muscle myosin II staining in fixed cells. M-band proteins, e.g., myomesin, are late assembling proteins to the mature myofibrils, presumably aiding thick filaments to become aligned side by side into A-bands. Nonmuscle myosin II proteins are absent from the mature myofibrils [4].

## Dynamics of Myofibrillar Proteins

The mechanism of sarcomere maintenance, collectively defined as the processes of sarcomeric protein synthesis, incorporation, and degradation, in a fully differentiated muscle cell is a topic of immense importance. There are a number of muscle diseases where the myofibrils exhibit various types of structural defects in their normal arrays, and we are uncertain how these changes occurred [6]. We suggest these abnormal alterations are due to some unknown assembly properties of the mutated sarcomeric protein. There have been several approaches to studying the exchange of sarcomeric proteins from the cytoplasmic pool with their counterparts in the existing myofibrils, processes often referred to as protein dynamics. These approaches have made important advances in understanding the mechanisms of synthesizing new myofibrils, or maintaining formed myofibrils. The formation of new myofibrils in embryonic and neonatal cardiac myocytes, and in skeletal muscle cells has been visualized by fluorescence imaging using isoform-specific, myofilament protein antibodies in fixed cells [2,4], and more importantly in living cells by the microinjection of fluorescently labeled sarcomeric proteins [1]. These studies have shown the requirement of precise regulation of myofilament protein gene expression and ordered integration of specific myofilament proteins into the contractile apparatus [3,5]. More recently, transfection and infection techniques have been used to express recombinant epitope-tagged myofilament proteins in embryonic cardiac and skeletal myocytes in vitro [7–9]. This, along with high resolution imaging techniques, has allowed the visualization of myofibrillogenesis, and mechanisms of myofilament protein isoform sorting in differentiating cardiac and skeletal myocytes [8,10–12].

In a global sense, myofilament protein turnover in adult muscle cells is well documented. Metabolic labeling studies have indicated that the half-lives of the major contractile proteins in the adult rat heart vary from 3 days for troponin I (TnI), 5 days for tropomyosin (Tm) and myosin, and up to 10 days for sarcomeric actin [13], which suggests that individual contractile proteins may turn over by different mechanisms. Interestingly, however, several studies of protein dynamics in live cells that superimposed on the processes of formation and maintenance of macromolecular complexes such as mature myofibrils is an active exchange of proteins between organized structures and cytoplasmic pools [9,11]. The time scale for this dynamic behavior is much shorter than protein synthetic half-lives, and occurs without affecting structural integrity.

### Dynamics of proteins present in Z-bodies and in Z-bands

**Figure 3:**
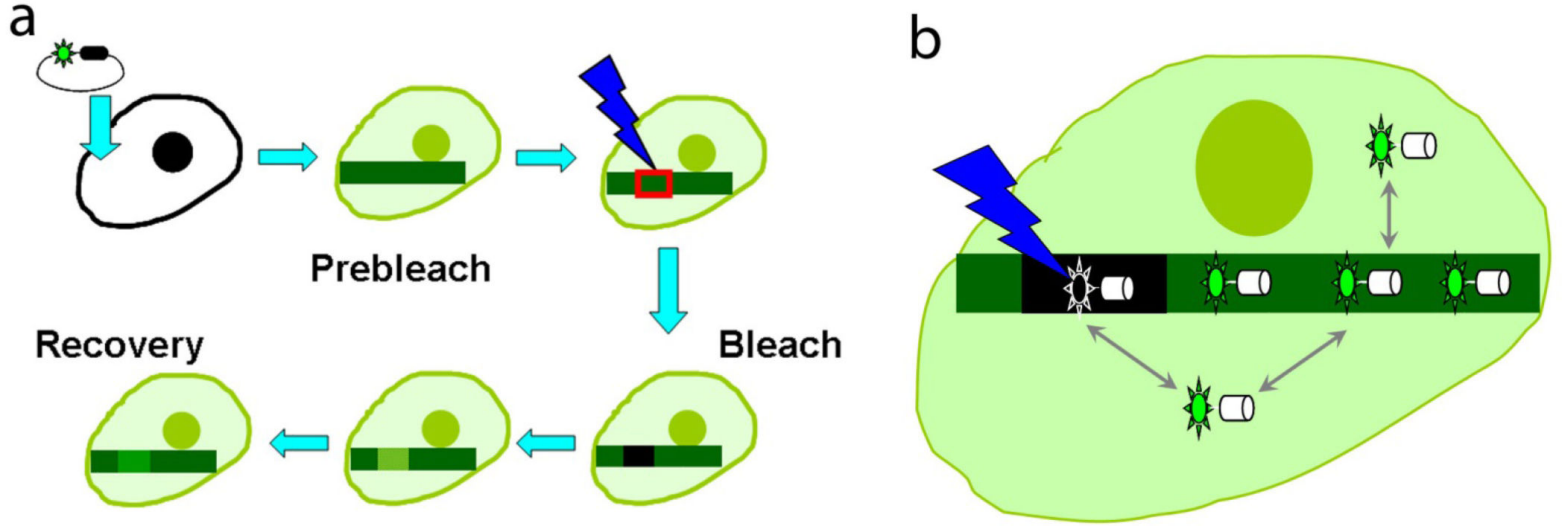
Fluorescence Recovery after Photobleaching (FRAP). FRAP is a useful technique to study the exchange of fluorescently tagged soluble sarcomeric proteins with their counterparts in the myofibril in living cells, processes referred to as protein dynamics. (a) In a FRAP experiment, the construct containing the studied protein linked to fluorescence protein was transfected and expressed in the target cells. FRAP experiment can be setup in three simple steps. First, pre-bleach Image was recorded using low intensity light as control. Second, an interested region was chosen and bleached using high intensity laser light. Third, a series of images were recorded using low light intensity to follow the fluorescence intensity change during recovery. (b) When the fluorescently tagged protein was expressed in the cell, most proteins were localized in the some special structure or area, but there are also free proteins in the cytoplasm pool. The protein in the special structure was in a dynamic exchange with the cytoplasm pool. By applying high intensity laser beam in a small area, the fluorescence protein in the region of interested was irreversibly bleached due to the photochemical destruction of the fluorophore, but the studied protein was intact and still in exchange with free fluorescently tagged proteins in the cytoplasmic pool. This results in the bleached protein coming out of the myofibril, and unbleached fluorescently labeled protein coming into the bleached region, and thus the fluorescence in recovered in the initially photobleached region.

Sarcomeres of cross-striated muscle vary among invertebrate species in length and in some protein constituents. However, all have a similar subunit arrangement of three major components: thin filaments, thick filaments, and Z-bands that each forms from multiple interactions among various proteins for producing and controlling contraction (Figure 1). During myofibrillogenesis, Z-bands assemble by the lateral fusion of the Z-bodies of premyofibrils and nascent myofibrils [4,8,9]. As the main focus of this editorial is on the dynamics of thin filament proteins especially tropomyosin, we will just summarize the results on the exchange of soluble Z-band proteins with their counterparts in the Z-bands of the mature myofibrils, processes referred to as protein dynamics, published from this laboratory. Wang et al. [9,10] using Fluorescence Recovery After Photobleaching (FRAP; see Figure 3 for the basis of this technique) technology to detect sarcomeric protein exchanges or dynamics demonstrated that seven different Z-band proteins exchanged independently of each other and that the exchange was independent of protein synthesis and molecular weight. Furthermore, the dynamics of the same proteins in z-bodies of premyofibrils were decreased in the Z-bands of the mature myofibrils. The decrease was suggested to be due to the incorporation of new binding proteins (e.g., titin and telethonin) in the forming Z-bands of nascent and mature myofibrils. These observations were extended to skeletal muscle cells in living zebrafish [2] where five different Z-bands were followed (actin, α-actinin, FATZ, myotilin, and telethonin). Their order of exchange was similar to the exchange of the same proteins in cultured quail skeletal muscle cells indicating that the behavior in culture conditions are comparable to that in cells in the living animal.

### Dynamics of proteins present in thin filaments

Thin filaments are composed of F-actin and several other proteins like tropomyosin (TM), troponin complex (TnT, TnC, and TnI), tropomodulin (Tmod), CapZ (Capping Z-band protein), etc. One end of thin filaments known as the barbed end (or fast growing end) in sarcomere is anchored within the Z-bands where they are capped with CapZ protein and cross-linked with a-actinin. The pointed ends (or slow growing ends) of thin filaments in sarcomere are capped by Tmod. Litttefield et al. [14] microinjected rho-actin into embryonic chick cardiomyocytes and demonstrated that rho-actin was incorporated at both the barbed and pointed ends of the thin filaments. Similar results were observed in skeletal myotubes. The results strongly suggest that although capped with capping proteins (CapZ or Tmod) both ends of thin filaments are transiently uncapped in living myocytes to allow monomer addition at the ends in vivo. They also transfected cardiomyocytes with an expression construct recovery after bleaching suggesting all the pointed ends are uncapped for part of the time. The authors with a tagged GFP (Green Fluorescent Protein)-Tmod and subsequently used FRAP technology. The FRAP results show about 100% recovery after photobleaching, which lead them to conclude that Tmod is dynamic at the pointed ends of thin filaments. Similarly using Cytochalasin D that inhibits actin incorporation at the barbed end, they concluded that CapZ is dynamic at the barbed ends of thin filaments.

Miichele et al. [15] determined the maintenance of thin filaments in adult cardiomyocytes under serum starved condition by infections with adenoviral vector containing epitope tagged tropomyosin (TM) and/or troponin I (TnI). Confocal microscopy and immunolocalization studies were used for evaluating the incorporation of tagged TM or tagged TnI into sarcomere. The replacement of endogenous TnI by tagged TnI was observed to be faster than the replacement of endogenous TM by tagged TM. Interestingly, tagged TnI was detected along the entire length of the thin filament but tagged TM was detected first at the pointed end (slow growing end) of the thin filament. The authors concluded that the nature of replacement of myofilament proteins is protein specific. They also provided a model for sarcomere maintenance, which they defined as the continual process of replacement of myofilament lattice with newly synthesized proteins in fully differentiated adult contractile cardiomyocytes.

The preceding studies using various state of the art technologies have provided several important/critical and novel information on the dynamics of the thin filament proteins in adult myocytes. These approaches, however, do not offer any information on the dynamics of various thin filament proteins at different stages of myofibrillogenesis. It is important to ask whether the dynamics of a thin filament protein for example, tropomyosin, remain the same or it may vary in a stagespecific manner. It is to be noted that the protein compositions in thin filament varies with the stage during myofibrillogenesis (Figure 2). Wang et al. [16] demonstrated that the dynamics of tropomyosin decreased as premyofibrils were transformed into mature myofibrils. In this study, the dynamics of two sarcomeric isoforms of tropomyosin designated as TPM1α and TPM1κ were determined in quail skeletal myotubes. TPM1α and TPM1κ are two alternatively isoforms of the TPM1 gene. Both TM isoforms having the same amino acid sequences except for exon 2 were found to be expressed in avian embryonic cardiac muscles [17]. In this study, Wang et al. [16] transfected quail skeletal muscle cells with Green Fluorescent Proteins (GFP) coupled to chicken TPM1α and chicken TPM1κ and compared their localizations in premyofibrils and mature myofibrils. Both isoforms of GFP-tropomyosin (TPM1α and TPM1κ) localized between the adjacent Z-bands of mature myofibrils marked by alpha-actinin when cotransfected with RFP-α-actinin. The dynamics of TPM1α and TPM1κ in myotubes were compared by using FRAP technique. The two TM isoforms were found to have a higher exchange rate in premyofibrils than in mature myofibrils.

The recovery profiles after bleaching of both TM isoforms in premyofibrils and mature myofibrils, suggest that tropomyosin exists in two states in the myofibril - as a dynamic mobile fraction and as a relatively static or immobile fraction. The FRAP data can best be fit to a two exponential equation, which predicts that the recovery of both tropomyosins is a two-step process with a fast and a slow component in both premyofibrils and mature myofibrils. In premyofibrils and in mature myofibrils, the half-time recoveries of the fast mobile fractions for both TM isoforms are about 10 s and the slow mobile fractions for both isoforms is about 200 s. The total mobile fractions (slow plus fast) of the two tropomyosin isoforms, however, are much higher in premyofibrils than in mature myofibrils. The slower exchange rates of both TM isoforms in mature myofibrils could be due to the addition of TM-binding proteins to the developing myofibrils. Although the exact sequence of binding is yet to be established, tropomyosin binds with several thin filament proteins for example, actin, troponin T, tropomodulin, and nebulin, in the course of formation of mature thin filaments during myofibrillogenesis. Findings from various laboratories suggest that nebulin is not connected with premyofibrils (or earlier stages of myofibrils) in skeletal muscle cells [18,19]. Nebulin is known to bind with actin, tropomyosin, troponinT, tropomodulin, and α-actinin, which in turn help to cross-link and stabilize thin filaments during myofibril assembly in skeletal muscle cells (Figure 2) [20]. The late assembly/connection of nebulin with myofibrils is substantiated by the immunofluorescence results with anti-nebulin and anti-tropomyosin antibodies [16]. The increase in the immobile fraction of both tropomyosin isoforms may reflect the final binding arrangements of the tropomyosin binding proteins, i.e., actin, troponinT, tropomodulin, and nebulin. In addition, Cap-Z that binds with nebulin [21], is present only in mature myofibrils [16]. Hence, the interaction of Cap-Z with nebulin may also render tropomyosin less dynamic in mature myofibrils.

Subsequently, Wang et al. [22] reported a FRAP studies of GFP-TPM1α and GFP-TPM1κ expressed in cultured avian cardiomyocytes. The recovery patterns and mobile fractions of the two TM1 isoforms show no significant difference in premyofibrils and in mature myofibrils in cardiomyocytes. The marked decrease in dynamics of TM as premyofibrils fuse to form mature myofibrils in skeletal muscle cells was not detected in cardiomyocytes [22]. The fact that the dynamics of the two TM isoforms are similar in premyofibrils and mature myofibrils in cardiomyocytes strengthen our working hypothesis that nebulin makes TM less dynamic in mature myofibrils in skeletal muscle. Pappas et al. [21] also reported that the dynamics of TM in chick skeletal myocytes was increased significantly when the expression of nebulin was knocked down by the gene-specific siRNA. The FRAP results of Pappas et al [21] confirmed the model proposed by Wang et al. [22] that nebulin is primarily responsible for the stabilization of tropomyosin in mature myofibrils in skeletal muscle cells. Nebulin attaches to thin filaments in mature myofibrils but is absent from the premyofibrils and nascent myofibrils (Figure 2). The dynamics of TM is higher in premyofibrils and nascent myofibrils where nebulin is not part of the thin filaments.

Our FRAP results show that the dynamics of tropomyosin are very similar, if not identical, in premyofibrils and mature myofibrils in cardiomyocytes [22] where the role of nebulin on the stability of myofibrils is not undisputed. Kazmierski et al. [23] reported that nebulin is expressed in mouse heart and skeletal muscle. However, the levels of nebulin transcript as well as its protein are much lower in hearts compared to skeletal muscle [24,25]. Knocking down the expression of nebulin in cultured rat cardiomyocytes by using gene specific sRNA, McEthinny et al. [26] claimed that nebulin is required for the proper assembly of thin filaments, maintenance of their lengths, and contractile function in cardiac myocytes. They also claimed that nebulin is involved in de novo myofibrillogenesis in rat skeletal muscle. To investigate the functional role of nebulin in vivo, Bang et al. [24] generated nebulin-deficient mice by using a Cre knock-in strategy. Lineage studies utilizing this mouse model demonstrated that nebulin is expressed uniformly in all skeletal muscles. Nebulin-deficient mice died within 8–11 d after birth, with symptoms including decreased milk intake and muscle weakness. Although myofibrillogenesis had occurred, skeletal muscle thin filament lengths were up to 25% shorter compared with wild type, and thin filaments were uniform in length both within and between muscle types. However, nebulin is not required for myofibrillogenesis but is essential for the maintenance of myofibril integrity during muscle contraction [24]. In addition, Bang et al. [24] reported that nebulin is expressed both in skeletal and cardiac muscle. However, nebulin was found to be expressed only in atrial cardiomyocytes and only in a small percentage of ventricular cardiomyocytes.

Will et al. [25] also created nebulin deficient mice and drew the similar conclusion on the role of nebulin in thin filaments in skeletal muscle. Witt et al. [25] stated that skeletal muscle have two nebulins per thin filaments, while cardiac muscle possess one nebulin per 50 thin filaments. This low ratio in cardiac muscle is inconsistent with the proposal that nebulin determines the uniform length of thin filaments in cardiac muscle. The results with nebulin knockout mice strongly support our conclusion that the dynamics of TM in cardiac premyofibrils and mature myofibrils are practically indistinguishable because of the fact that nebulin is not involved in stabilizing thin filaments in cardiomyocytes [22].

## Conclusion

The theory that nebulin is not involved in cardiac myofibril organization is further supported by the fact that several missense mutations in nebulin have been implicated in nemaline myopathy in skeletal muscle [27,28]. Thus far no nebulin mutations have been reported to be associated with any cardiomyopathies in humans.

Nebulette is the analogous protein of the skeletal muscle’s nebulin in cardiomyocytes. Nebulette (M.W. 107 kD) compared to nebulin (~ 800 kD) is much shorter and does not cover the total length of actin filament in heart. Nebulette, however, may participate in both structure and the function of the Z-band; targeted disruption of nebulette expression may alter cardiac myofibril assembly and function [29]. Several missense mutations in nebulette have been implicated in various dilated cardiomyopathies in humans [30,31]. The latter observations support the hypothesis that nebulette plays a critical role(s) in cardiac contractility. To the best our knowledge, there is no report on the nebulette knock out mouse, which is crucial to draw a definitive conclusion that nebulette plays a critical role in cardiac myofibrillogenesis and cardiac contractility.

As far as the dynamics is concerned, tropomyosin is significantly less dynamic in mature myofibrils of skeletal muscles, where nebulin is an integral part of the thin filament, than in premyofibrils where nebulin is not present. Due to the absence or low level of nebulin in cardiomyocytes, tropomyosin is more dynamic in both premyofibrils and matured myofibrils in cardiomyocytes than in matured myofibrils in skeletal muscle cells [22]. Our FRAP results [16,22] along with the results from nebulin knockout mice [24] strongly support our working hypothesis that nebulin after its association with the thin filaments in matured myofibrils in skeletal muscle, renders tropomyosin less dynamic.

There are a number of skeletal muscle diseases whereby the normal structure of myofibrils is disrupted by the presence of mutated or truncated sarcomeric proteins [6]. Our work on truncated proteins in cultured skeletal muscle cells has demonstrated the dramatic effects on the malformation and maintenance of mature myofibrils [10,32]. Heart disease is the number one cause of death worldwide. A significant aspect of the problem is found in cardiomyopathies, heart disease that arises from dysfunction of the heart muscle [33]. With increasing frequency, genetic analysis is uncovering mutations in sarcomeric proteins as prominent causes of hypertrophic cardiomyopathy (HCM) and dilated cardiomyopathy (DCM), the two most prevalent forms of cardiomyopathy [34]. The former leads to an increase in ventricular muscle mass and impaired relaxation and the latter are characterized by dilated ventricle and impaired contractile function. The unifying link between these opposing outcomes is the role of sarcomeric and sarcomere-associated protein mutations. In some cases two different mutations in the same protein can lead in one instance to DCM and in the other to HCM. A significant challenge in the field is to determine how the mutated sarcomeric protein disrupts the normal function of the myofibril to produce these varying phenotypes. We predict that analyses of the mutant proteins in living muscle cells will reveal changes in the dynamics of the mutant protein in the context of the network of the many interacting proteins that form the sarcomere. These studies will complement studies focused on isolated mutant proteins and add significantly to the goal of gaining fundamental insights into the molecular bases of muscle diseases.
